# Tumoral calcinosis (Teutschlander disease) in a dialysis patient

**DOI:** 10.4103/0971-4065.43692

**Published:** 2008-07

**Authors:** P. Binnani, V. Aggarwal, M. M. Bahadur, N. Fulara

**Affiliations:** Department of Nephrology, Jaslok Hospital and Research Centre, Mumbai - 400 026, India; 1 Department of Medicine, Jaslok Hospital and Research Centre, Mumbai - 400 026, India

**Keywords:** Aluminum intoxication, calciphylaxsis, tumoral calcinosis

## Abstract

Tumoral calcinosis is an uncommon and severe complication of hemodialysis therapy. It is generally associated with the presence of the high serum calcium-and-phosphorus product. We report here a case of a patient on hemodialysis who presented with progressively increasing, multiple, tumor-like, subcutaneous swellings. These are rare manifestations of extraosseous calcification in uremic patients that are termed as tumoral calcinosis. A 25 year-old male presented with multiple, nodular, painful, cutaneous swellings all over his body that had been progressively increasing over the last four years. He was a known case of chronic glumerulonephritis who was on regular hemodialysis. The patient was investigated and diagnosed as having tumoral calcinosis and was treated with a low calcium dialysate of pure reverse osmosis water.

## Introduction

Tumoral calcinosis is an uncommon and severe complication of hemodialysis therapy.^1^–^3^ The genesis of tumoral calcification has been attributed to high calcium and phosphorus products and/or advanced secondary hyperparathyroidism after long-term dialysis. The patient presented with huge, tumor-like, subcutaneous deposits (tumoral calcinosis) and calciphylaxsis (uremic arteriolopathy), which seldom occur in conjunction. We report here a case of severe secondary hyperparathyroidism with metabolic tumoral calcinosis.

## Case Report

A 25 year-old male, hailing from Iraq, presented with multiple, nodular, painful, cutaneous swellings all over his body that had been progressively increasing over the last four years. His primary renal disorder was chronic glomerulonephritis and he was on hemodialysis since January 2000. In March 2000, he received a renal allograft from an unrelated donor, which failed after two months due to acute rejection; a graft nephrectomy was subsequently done. Thereafter, he was on hemodialysis two times a week, each session for two hours only. He had pulmonary tuberculosis in December 2000, for which he took antituberculosis medications for nine months. He was also diagnosed to be hepatitis C-positive (chronic active hepatitis) in 2003, and was treated with interferon which was stopped due to pancytopenia. The swellings started 3½ years ago, progressively appearing on the neck, trunk, and the extremities. Some of these swellings ulcerated spontaneously with a foul-smelling discharge. Biopsy of the lesion in Iraq revealed only degenerative changes and necrosis with no evidence of malignancy (stain for calcium was not done). He also complained of abnormal gait and recurrent high-grade fever.

**Fig. 1:**
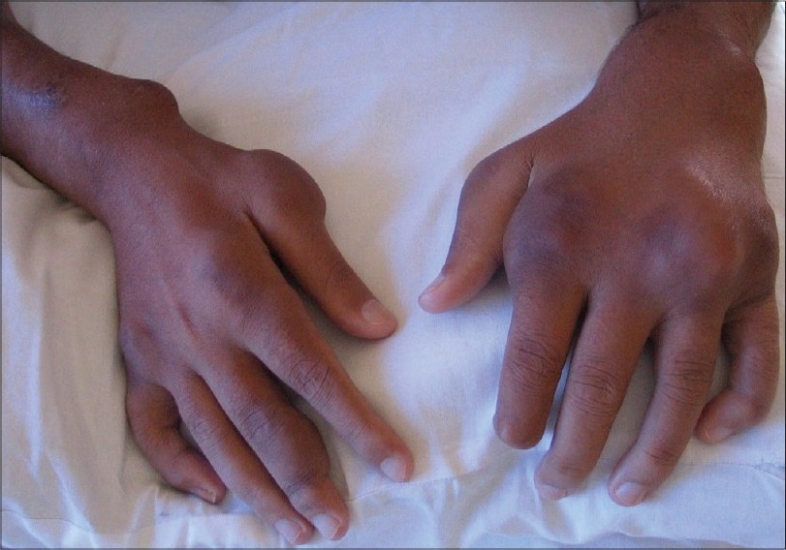
Subcutaneous multilobular masses

He was admitted for the evaluation of these complaints at our Center in August 2007. Physical examination revealed subcutaneous, multilobular masses ranging from 2 × 2 cm to 10 × 15 cm, mainly periarticular, with a reduced range of movements at these joints [Fig. 1]. He had voilaceous, indurated, and painful nonhealing ulcers on his hands and feet with a bilateral foot drop. Investigations revealed: hemoglobin 5.8 g/dL, peripheral blood leukocyte count 4280/mm^3^ and platelet count 22 × 10^4^/µl, BUN 115 mg/dL; creatinine 15.3 mg/dL; calcium 9.1 mg/dL; phosphorus 9.8 mg/dL; alkaline phosphatase 378 IU; uric acid 9.1 mg/dL; erythrocyte sedimentation rate 140 mm/L. Rheumatoid factor and antinuclear antibody were absent. Intact PTH was 1315 pg/ml (12–72 pg/ml); 25-hydroxy-vitamin D_3_ 38.4 nM (23–123 nmol/L); serum aluminum level was 232.6 µg/L (< 40 ug/L).

**Fig. 2:**
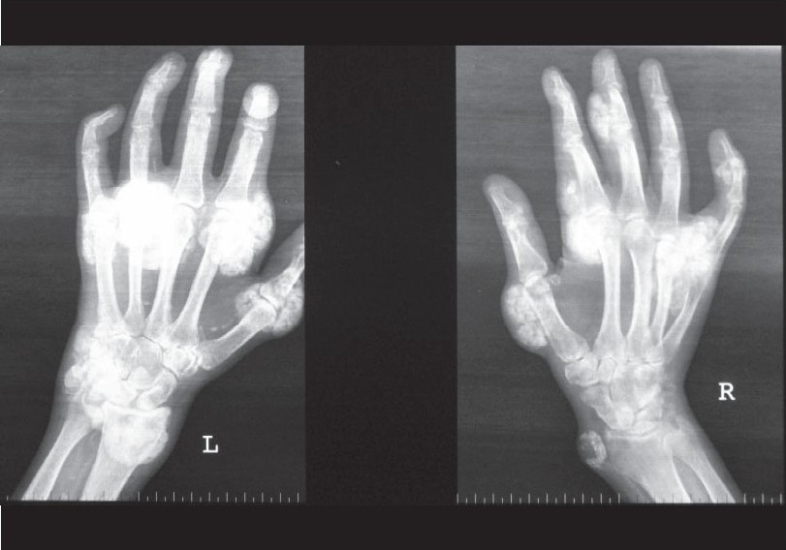
X-ray of the hand

**Fig. 3:**
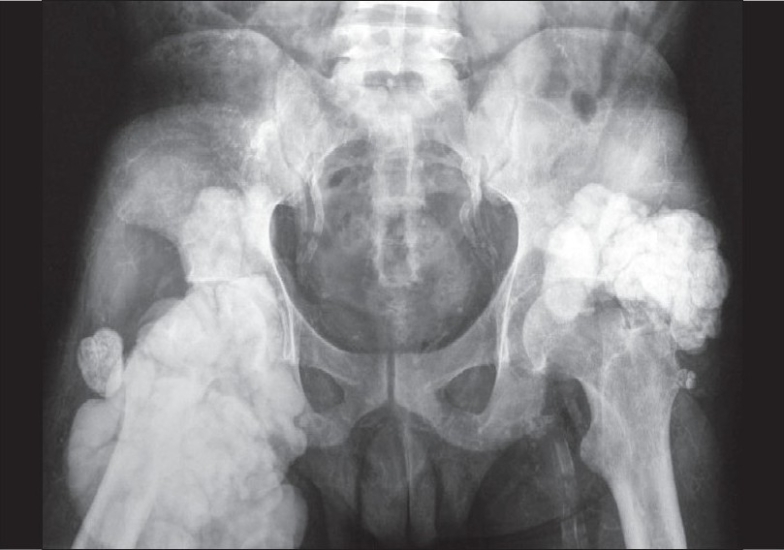
X-ray of the pelvis with both hips

**Fig. 4:**
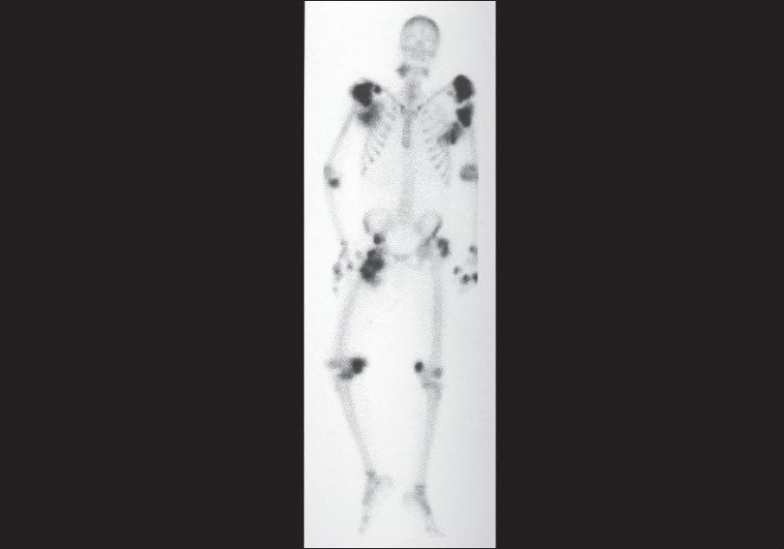
Radioisotope bone-scan

An X-ray of the joints revealed gross, periarticular, soft tissue calcification with vascular calcification [Figs. 2 and 3]. USG of the abdomen revealed splenic vessel calcification and bilateral, small, shrunken kidneys with dystrophic specks of calcification. Ultrasound of the nodular lesion was suggestive of calcified subcutaneous masses (solid and cystic). A radioisotope bone-scan revealed multiple areas of abnormal soft tissue uptake suggestive of ectopic calcification [Fig. 4]. A parathyroid scan did not pick up any parathyroid adenoma. A CT scan of the spine showed paravertebral, calcified masses with fluid calcium levels suggestive of heterotopic soft tissue calcification. The patient had concentric left ventricular hypertrophy with an ejection fraction of 55% on 2D echocardiography. Electromyography and nerve conduction studies showed mixed (demyelinating and axonal) sensorimotor radiculoneuropathy. Causes for the patient's recurrent fever were investigated, but all cultures were negative.

A diagnosis of tumoral calcinosis was made and the patient was started on alternate day, four hours' dialysis using a low-calcium dialysate of pure reverse osmosis water. A noncalcemic phosphate binder, Sevelemer, 800 mg three times a day, was added to reduce the levels of calcium-and-phosphorus product. As his deferoxamine test for aluminum toxicity was positive (high baseline with doubling of serum aluminum levels on the deferoxamine test), he was started on desferal chelation for aluminum toxicity. His symptoms of fever and painful joints improved after eight sessions of dialysis at our Center, after which he opted to continue further treatment in his own hometown.

## Discussion

Tumoral calcinosis is an uncommon ectopic calcification syndrome. Tumoral calcinosis and calciphylaxis are complications associated with the high serum calcium-and-phosphorus product in end stage renal failure patients who often have secondary or tertiary hyperparathyroidism.^4^ Persistently elevated calcium-and-phosphorus product is the major contributing factor in the development of tumoral calcinosis, although secondary hyprerparathyroidism, adynamic bone disease, hypermagnesemia, vitamins D and K overload, aluminum intoxication, metabolic alkalosis, and tissue injury are also implicated.^5^ The common sites are the elbow, hip, shoulder, and the hand with reduced joint mobility and arthralgia; compression of the adjoining structures may give rise to neurovascular symptoms. There are reports of tumoral calcinosis presenting with signs of systemic inflammation, leading to fever and constitutional symptoms (as in this case) mimicking infection.^6^ Calciphylaxsis occurs in 1–4% of dialysis patients, and is characterized by cutaneous ischemia, which ulcerates with voilaceous, indurated, and painful areas.^7^

Management of tumoral calcinosis is often difficult and involves dietary phosphate restriction, noncalcemic phosphate binders and intensification of dialysis treatment using a low-calcium dialysate, parathyroidectomy in patients with high PTH levels due to tertiary hyperparathyroidism, and surgical excision of the mass. Tumoral calcinosis resolves after successful transplantation.^8^ Some reports describe biphosphonate use to prevent a systemic inflammatory response due to cytokine release from osteoclastic activity.^6^ Sodium thiosulfate has been used for tumoral calcinosis in the absence of hyperparathyroidism or in the presence of a high calcium-and-phosphate product. It has been claimed that sodium thiosulfate inhibits the formation and favors solubility and the mobilization of calcified masses.^9^ Aluminum intoxication has also been described to contribute in the development of tumoral calcinosis.

## Conclusion

Tumor calcinosis is an uncommon ectopic calcification syndrome and is a rare complication of chronic renal failure.
